# Retrospective quality assurance audit of Lateral Cephalometric Radiographs at postgraduate teaching hospital

**DOI:** 10.12669/pjms.36.7.2796

**Published:** 2020

**Authors:** Anum Khan, Muhammad Qasim Javed, Rabia Bilal, Rahul N Gaikwad

**Affiliations:** 1 Dr. Anum Khan, MSc, Clinical Demonstrator, Islamic International Dental College and Hospital, Islamabad, Pakistan; 2 Dr. Muhammad Qasim Javed, FCPS, Assistant Professor, Department of Conservative Dental Sciences and Endodontics, College of Dentistry, Qassim University, Buraydah, Saudi Arabia; 3 Dr. Rabia Bilal, FCPS, Associate Professor, Dept. of Orthodontics, College of Dentistry, College of Dentistry, Qassim University, Buraydah, Saudi Arabia; 4 Dr. Rahul N Gaikwad, PhD, Associate Professor, Department of Community Dentistry and Oral Epidemiology, College of Dentistry, Qassim University, Buraydah, Saudi Arabia

**Keywords:** Audit, Lateral Cephalometric Radiograph, Quality assurance, Radiation protection

## Abstract

**Objective:**

The objective of our audit was to assess the quality of lateral cephalometric radiographs by investigating the percentage of lateral cephalometric radiographic images that satisfied the good quality standards.

**Methods:**

The standard-based retrospective audit was conducted at Riphah International University, Pakistan, from April to September 2018. The sample size was 50 radiographs that were randomly selected from the radiographs taken over one year. The radiographs were graded according to the standards set by the National Radiation Protection Board by two evaluators after the necessary calibration. Moreover, the percentages of images that met the criteria set by the Royal College of Surgeons of England were identified. The data analysis was carried out by the SPSS software version 23.

**Results:**

Out of the 50 radiographs, thirty-one were Grade-I, sixteen were Grade-II and three radiographs were Grade-III. Furthermore, out of the criteria set by Royal College, one criterion met the 100% standard that was correct head positioning. Less frequent errors were comprised of poor visibility of soft tissue structures (16%), teeth not properly occluded (14%), Incorrect positioning of labels (12%), Sella and Nasion not visible (8%). According to the results of the quality assurance audit, the radiographs fell short of the required standards.

**Conclusion:**

Quality assurance by periodic auditing is important to yield radiographs with maximum diagnostic value, minimal errors, and avoid unnecessary radiation exposure by repeat radiographs. Recommendations were made for the formulation and implementation of comprehensive radiation protection regulations, at all the Dental institutes of Pakistan.

## INTRODUCTION

A Lateral cephalometric radiograph (LCR) is a fundamental orthodontic diagnostic tool that is essential for the diagnosis of skeletal, dental and soft tissue anomalies of facial structures. It also monitors the progress of skeletal and dental changes during treatment, helps to assess the growth along with aiding as pre-treatment record and post-treatment comparison template.^1^,^2^ The radiation exposure for these radiographs is comparatively lower than the three-dimensional imaging and proves to be an effective dose-efficient diagnostic and monitoring tool.^3^ Hofrath and Broadbent presented lateral cephalogram as a standardized technique using a high powered x-ray machine and a head holder called cephalostat.^4^ In order to keep it standardized they made few guidelines for taking lateral cephalograms for every patient. The patient is positioned within the cephalostat using adjustable bilateral ear rods placed within each external auditory meatus. The midsagittal plane of the patient is vertical and parallel to the film plane and perpendicular to the x-ray beam. The patient should be with relaxed lip and mentalis muscle and bite in centric occlusion.

As per the ‘Ionizing Radiations (Medical Exposure) Regulations’ (IRMER 2017), intraoral and extraoral radiographs should be consistent in generating a high quality radiographic images to limit the radiation exposure of patients to doses as low as reasonably practicable (ALARP).^5^ Therefore, to minimize the harmful effects of radiation, the use of lateral cephalograms should be judicial and the standardization protocol must be followed. Since the cephalometric analysis requires landmark identification and interpretation of varying angular and linear measurements for the diagnosis and treatment planning, the errors should be minimized to avoid any unnecessary repetition of radiographs.^6^,^7^ Quality Assurance (QA) plays an essential role in optimizing the diagnostic value by minimizing projection, magnification, exposure, and other radiographic errors and aiding in accurate interpretation.^8^-^10^

The Royal College of Surgeons of England (RCSEng) formulated guidelines for standardization to ensure good quality radiographs.^11^ The guidelines suggested 12 parameters to be the prerequisite for high quality lateral cephalograms.^11^,^12^ The objective of the current audit was to assess the quality of lateral cephalometric radiographs by investigating the percentage of radiographic images that satisfied the good quality standards and also to determine the criteria of the lateral cephalometric radiographs that did not attain the set standards.

## METHODS

This retrospective audit was conducted at the Department of Orthodontics, Riphah International University, Pakistan, from April to September 2018. The ethical approval for conducting an audit was obtained from the institutional ethical review board (Approval No. IIDC/IRC/2017/10/005, dated Oct. 9, 2017). The sample size for the first cycle of clinical audit comprised of 50 pretreatment lateral cephalometric radiographs that were randomly selected from the Radiographs requested by the Department of Orthodontics, over one year from 1^st^ April 2017 to 31^st^ March 2018. Before commencing audit calibration was done among the evaluators to exclude inter-evaluator bias and enhance inter-evaluator reliability. Initially, ten lateral cephalometric radiographs that were not included in the audit were individually assessed by two evaluators, both with clinical experience of more than five years. The evaluators graded the films as per the system of grading developed by the National Radiation Protection Board.^13^ The inter-evaluator reliability was calculated by Cohens Kappa and was found to be satisfactory. Subsequently, the patients’ medical record numbers were coded from 1 to 50. The hard copies of LCRs’ were analyzed on the radiographic illuminator in the darkroom by two experienced clinicians, individually. In case of a difference of interpretation, the opinion of the consultant orthodontist was taken to reach consensus. The evaluation process of the quality of LCR was comprised of identifying the percentages of the LCRs that met the parameters set by the Royal College of Surgeons of England.^11^ Data were recorded on a standardized data collection form adapted from the methodologies for clinical audit in Dentistry laid down by the Royal College of Surgeons of England, to add one additional variable that is Removable appliance removed, thereby, making a total of 13 point criteria. Following variables were included a) Correct head position b) Important structures centered on the film c) Soft tissues visible d) Teeth in occlusion e) Good contrast f) Name and hospital number recorded g) Label not obscuring radiograph h) Nasion identifiable i) A-point identifiable j) B-point identifiable k) Sella identifiable l) Incisors visible and their angulation measureable m) Removable appliance removed.

Later, the radiographs were graded according to the grading criteria set by NRPB (Table-I).^13^ Descriptive statistics were analyzed by utilizing the Statistical package for social sciences SPSS software version 23 (IBM Corp,32 Armonk, N.Y., US).

**Table-I:** The system of grading by the National Radiographic Protection Board (NRPB)^13^

Rating	Quality control	Target
1	Excellent- no errors of processing or positioning or exposure	Not less than 70%
2	Acceptable- some processing errors, exposure or positioning but which still allow diagnostic information to be obtained	Not greater than 20%
3	Unacceptable- errors render the film diagnostically useless	Not greater than 10%

## RESULTS

A sample of 50 lateral cephalometric radiographic films was analyzed. Out of the 50 radiographs, 31 radiographs were Grade-I, 16 radiographs were Grade-II and 3 radiographs were Grade-III. Considering the aforementioned results, the results of the first audit cycle fell short of the required standards, as depicted in Table-II.

**Table-II:** Results of the first cycle of data collection for lateral cephalometric radiographs, as compared to the target percentages.

Grading	Percentage NRBP Standard	Percentage (n) Current Study
Grade-I	Not less than 70%	62% (31)
Grade-II	Not greater than 20%	32% (16)
Grade-III	Not greater than 10%	6% (3)

**Fig.1:**
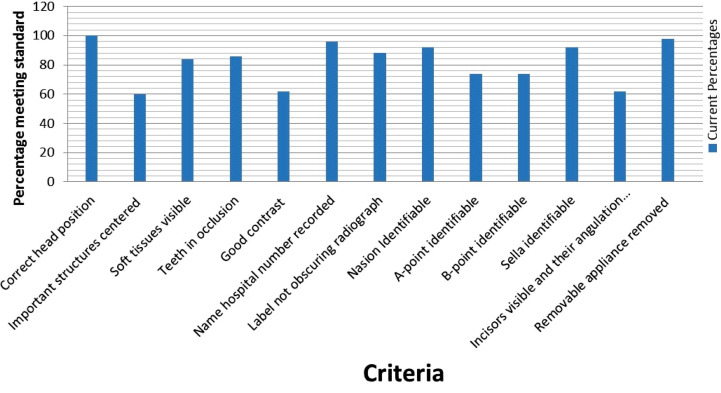
The percentage of criteria meeting the standard.

Furthermore, out of the 13 criteria, only one of the criteria met the 100% standard that was correct head positioning. Less frequent errors were comprised of poor visibility of soft tissue structures (16%), teeth not properly occluded (14%), Incorrect positioning of labels (12%), Sella and Nasion not visible (8%). The details of the results are depicted in Table-III and Fig.1.

**Table-III:** Criteria that fulfilled/not fulfilled the prerequisite for the good quality lateral cephalometric radiographs.

Criteria	Criteria Fulfilled % (n)	Criteria not Fulfilled % (n)
Correct head position	100% (50)	0% (0)
Important structures centred on film	60% (30)	40% (20)
Soft tissues visible	84% (42)	16% (8)
Teeth in occlusion	86% (43)	14% (7)
Good Contrast	62% (31)	38% (19)
Name and hospital number recorded	96% (48)	4% (2)
Label not obscuring radiograph	88% (44)	12% (6)
Nasion identifiable	92% (46)	8% (4)
A-Point Identifiable	74% (37)	26% (13)
B-Point Identifiable	74% (37)	26% (13)
Sella Identifiable	92% (46)	8% (4)
Incisors visible and their angulation measureable	62% (31)	38% (19)
Removable appliance removed	98% (49)	2% (1)

## DISCUSSION

Despite the ionizing radiations of the radiographs, radiology is the most important and common tool for diagnosis and treatment planning in medicine and dentistry. This brings in the importance of the quality of such a universally acceptable tool. Clinicians require a radiograph that is reliable in giving information about the disease and should be reproducible. The failure to serve this purpose will lead to the repetition of the radiograph hence increasing the cost, radiation exposure, and lack of diagnosis of the actual disease. This outweighs the advantages of such a simple yet effective tool. The good quality radiograph therefore single handedly is the most important pre-requisite for the radiographic diagnosis.^14^,^15^ The importance of lateral cephalogram in orthodontics is magnanimous. A good quality lateral cephalogram is important to detect underlying pathology, identifying the anatomical landmarks and accurate linear and angular measurements of skeletal, dental, and soft tissue analysis. All the important landmarks and structures must be identifiable and reproducible on the film to ensure their accurate utilization in the diagnosis and treatment planning phases.^12^

This audit was done to assess the quality of the lateral cephalograms. About 62% percent of radiographs were classified as Grade-I, 32% as Grade-II, and 6 % as Grade-III requiring repeat radiograph that was well below the standards set by NRPB. The radiographs that were categorized as Grade-III were those without patient name/hospital number and one radiograph in which removable appliance was not removed. This indicates that an improvement in radiographic quality is necessary to obtain good quality diagnostic radiographs and limiting the patients’ exposure to extra radiation.

The review of the audit literature reveals that other than teeth being in occlusion, good contrast, correct head position and centralization of structures, most important identifiable landmarks on the lateral cephalogram which ensure a good quality radiograph are point A and B, sella and nasion, upper and lower incisors and the soft tissue outline.^16^ The results of this study generated suboptimal results with only one out of the set 13 criteria points meeting the 100% standard. Although all the lateral cephalograms were taken in correct head position and most of the patients had their appliances removed but the structures were not centralized and contrast was not good in a large percentage of samples. Likewise, the visibility of soft tissue and occlusion was also compromised. The frequently prevalent error in the radiographs was the unclear visibility of upper and lower incisors, making the measurement of their angulation difficult. Upper and lower incisors must be clearly visible so their inclination and angulation can be measured accurately before the treatment starts and to evaluate the treatment outcome postoperatively.^17^ It is one of the tools to guide the orthodontist towards extraction or non-extraction orthodontic therapy.^18^The unclear apices of incisors may also be a manifestation of the poor contrast of the hard copy of the radiographic image. Similarly, Point ‘A’ and Point ‘B’ were not clearly visible in many radiographs. Point ‘A’ is defined at the deepest point on the curvature of the maxillary bone, lying between the anterior nasal spine and alveolar crest of the upper central incisor. This point determines the saggital relationship between the maxilla and cranial base and is used for anteroposterior measurements.^19^ Point ‘B’ that determines the saggital relation of the mandible to the cranial base is the most posterior point from infra dentate to pogonion. Exposure settings that have been set higher than the required value produce a darker image while a lower setting creates a paler image. Also, the kV setting of the X-ray machine affects image contrast. Poor contrast is the result of kV value being set higher or lower than the manufacturer’s recommended values.^20^ Such errors can make it difficult to identify the bony structures like point “A” and “B”.

Images being too light or too dark can also be the consequence of miscalculations during the processing procedure. Processing requires a dark room with no light leaks or a daylight loader with tightly sealed armholes to ensure light proofing. The daylight loader being currently used at the institute has minor tears in the arm holes, resulting in light leaks. Moreover, the overall temperature of the room needs to be about 17 degrees which is optimum for processing the films. The developing solution weakens over time and needs to be changed after a maximum of 10-14 days. Similarly, films that have been stored for prolonged times expire and produce dark, foggy images.^21^

The intercuspal position is the ideal position for orthodontic assessment unless the patient presents with mandibular displacement (>2mm). Hence, the Cephalometric radiograph should be taken in this position to generate an image with teeth in occlusion. Many radiographs presented with teeth out of occlusion, which may lead to incorrect analysis and treatment planning.^11^

The outcome of the audit was presented to the head of the institute and the following recommendations were made

- Training of the radiology team to be competent in the use of radiographic equipment, radiation protection and patient communication.
- Making sure the patient is not wearing any jewellery or removable appliances during exposure.
- Introduce the digital cephalometry machine with image enhancing software and electronic transfer of radiographs to the orthodontics department.
- Changing developing/ fixing solutions regularly according to the manufacturer’s recommendation and storing films properly.
- Adjustment of exposure, processing time and temperature
- Darkroom and daylight loader integrity should be checked and maintained
- Periodic audits after two years should be carried out^9^

### Strengths of the study

The strength of this audit is that internationally accepted standardization guidelines formulated by Royal College of Surgeons of England and grading system developed by National Radiation Protection Board were utilized in combination, for the quality assessment of lateral cephalometric radiographs.^11^,^13^

### Limitations of Study

This study highlighted the areas where improvement was required but there was a limitation. The limitation of the study was a relatively small sample size.

## CONCLUSIONS

The quality assurance by periodic auditing is important to yield results with maximum diagnostic value, minimal errors, and avoid unnecessary radiation exposure by repeat radiographs. In the current audit, one out of 13 parameters fulfilled the 100% criteria standard. The outcomes of the study highlight the significance of conducting regular quality assurance radiographic audits for assessing and monitoring the quality of the lateral cephalometric radiographs.

### Authors’ Contribution:

**AK** conceived, designed and did the data collection

**MQJ** did statistical analysis, write up & editing of the manuscript

**RB** did data collection and manuscript writing.

**RNG** did the review and final approval of the manuscript

**MQJ** is responsible and accountable for the accuracy or integrity of the work.
